# 

**Published:** 1966-10

**Authors:** 


### *Contents*

Page

THE ADVANCE OF GASTROENTEROLOGY ... *J. M. Naish* 58

INTIMATIONS OF SENILITY ........................ *T. W. Lloyd* 65

EXPERIENCES IN THE CONTROL OF LONG TERM ANTICOAGULENT TREATMENT ............... *A. B. Raper* 68

THE HISTORY OF JAUNDICE IN BRISTOL ... *Bimla Arora* 73

